# Author Correction: Ensembles of data-efficient vision transformers as a new paradigm for automated classification in ecology

**DOI:** 10.1038/s41598-023-32258-4

**Published:** 2023-04-17

**Authors:** S. P. Kyathanahally, T. Hardeman, M. Reyes, E. Merz, T. Bulas, P. Brun, F. Pomati, M. Baity-Jesi

**Affiliations:** 1 Eawag, Überlandstrasse 133, 8600 Dübendorf, Switzerland; 2 WSL, Zürcherstrasse 111, 8903 Birmensdorf, Switzerland

Correction to: *Scientific Reports* https://doi.org/10.1038/s41598-022-21910-0, published online 03 November 2022

The original version of this Article contained errors, where the Ensembles of Data-efficient image Transformers (EDeiTs) had a lower performance than stated.

After publication, the authors noticed a bug in the evaluation of the EDeiT models, where different models in the same ensemble were seeing different images, (always from the same class), which artificially increased the test performance. This bug was only present in the script for EDeiTs, all other models (single models and ensembles), were unaffected. The main conclusion of the paper, that one can use EDeiTs to obtain well-performing models avoiding parameter tuning, is still valid, since the performances are always similar to the previous state-of-the-art (SOTA). Errors have been corrected in the Abstract, the Results, the Discussion, Figure 1 and its caption, Figure 2, Table 1, and the Supplementary Information file. The original Figures 1 and 2, and the original Supplementary Information file are provided below. In the Results, the subheading ‘Arithmetic versus geometric averaging for ensembling’ and Table 2 have been removed. Consequently, the following references have been removed from the reference list and subsequent references have been renumbered in the text.

34. Alkoot, F. & Kittler, J. Experimental evaluation of expert fusion strategies. *Pattern Recogn. Lett.* **20**, 1361–1369. 10.1016/S0167-8655(99)00107-5 (1999).

35. Kittler, J., Hatef, M., Duin, R. & Matas, J. On combining classifiers. *IEEE Trans. Pattern Anal. Mach. Intell.* **20**, 226–239. 10.1109/34.667881 (1998).

37. Mi, A. & Huo, Z. Experimental comparison of six fixed classifier fusion rules. *Proc. Eng.* **23**, 429–433. 10.1016/j.proeng.2011.11.2525 (2011).

As a result of the errors, the Abstract,

“We overcome this limitation through ensembles of Data-efficient image Transformers (DeiTs), which not only are easy to train and implement, but also significantly outperform the previous state of the art (SOTA). We validate our results on ten ecological imaging datasets of diverse origin, ranging from plankton to birds. On all the datasets, we achieve a new SOTA, with a reduction of the error with respect to the previous SOTA ranging from 29.35% to 100.00%, and often achieving performances very close to perfect classification. Ensembles of DeiTs perform better not because of superior single-model performances but rather due to smaller overlaps in the predictions by independent models and lower top-1 probabilities. This increases the benefit of ensembling, especially when using geometric averages to combine individual learners. While we only test our approach on biodiversity image datasets, our approach is generic and can be applied to any kind of images.”

now reads:

“We overcome this limitation through ensembles of Data-efficient image Transformers (DeiTs), which we show can reach state-of-the-art (SOTA) performances without hyperparameter tuning, if one follows a simple fixed training schedule. We validate our results on ten ecological imaging datasets of diverse origin, ranging from plankton to birds. The performances of our EDeiTs are always comparable with the previous SOTA, even beating it in four out of ten cases. We argue that these Ensemble of DeiTs perform better not because of superior single-model performances but rather due to smaller overlaps in the predictions by independent models and lower top-1 probabilities, which increases the benefit of ensembling.”

In the Introduction,

“We show that while the single-model performance of DeiTs matches that of alternative approaches, ensembles of DeiTs (EDeiTs) significantly outperform the previous SOTA, both in terms of higher accuracy and of better classification of minority classes (*i.e.* rare species). We see that this mainly happens because of a higher disagreement in the predictions, with respect to other model classes, between independent DeiT models. Finally, we find that while CNN and ViT ensembles perform best when individual learners are combined through a sum rule, EDeiTs perform best when using a product rule.”

now reads:

“We show that while the single-model performance of DeiTs matches that of alternative approaches, ensembles of DeiTs (EDeiTs) achieve very good performances without requiring any hyperparameter tuning. We see that this mainly happens because of a higher disagreement in the predictions, with respect to other model classes, between independent DeiT models.”

In the Results, under the subheading ‘A new state of the art’,

“As shown in Fig. 1, the error rate of EDeiTs is drastically smaller than the previous SOTA, across all datasets.”

now reads:

“As shown in Fig. 1, the error rates of EDeiTs are sometimes close to or even smaller than those of previous SOTA.”

**Figure 1:**
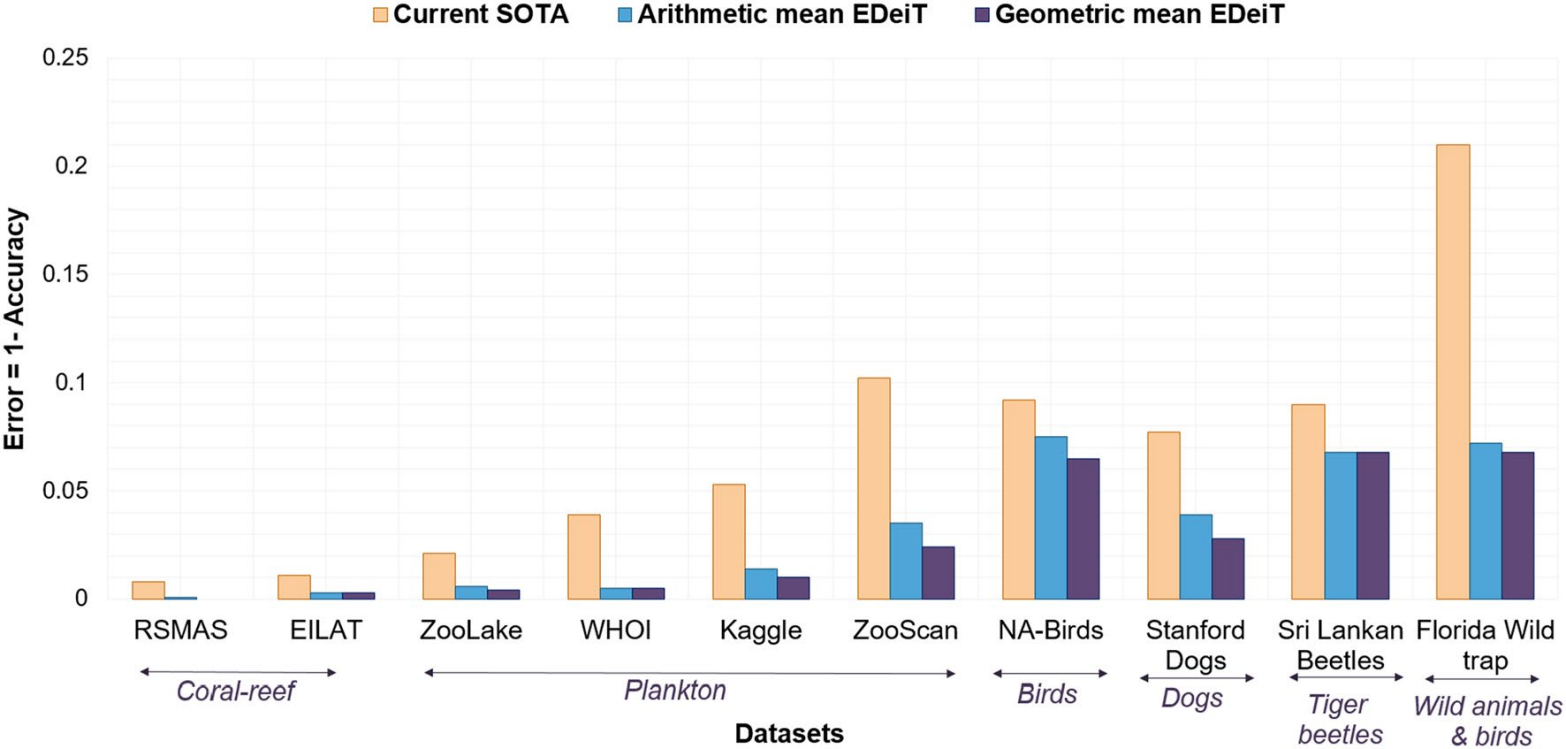
Comparing EDeiTs to the previous SOTA. For each dataset, we show the error, which is the fraction of misclassified test images ($1-accuracy$). The error of the existing SOTA model is shown in orange. For the ensembles of DeiTs, we show two ways of combining the individual learnings: through arithmetic (blue) and geometric (purple) averaging. The purple bar for RSMAS is absent because *all* the test examples were classified correctly by the EDeit with geometric averaging. Independent of the ensembling rule, our models outperform current SOTA models on a consistent basis.

Furthermore, under the subheading ‘Ensemble comparison’ of the same section,

“While all ensembled CNNs perform similarly to each other $F1-score\le 0.924$ ensembled DeiTs reach an almost perfect classification accuracy (with the F1-score reaching 0.983). With arithmetic average ensembling, we have 15 misclassifications out of 2691 test images, 14 of which on classes not associated to a specific taxon classes (see Supplemental Information, SI (See Footnote 1)). With geometric averaging, the performance is even higher with only 10 out of 2691 misclassified test images.”

now reads:

“The CNN family reaches a maximum $F1-score\le 0.920$ for ensemble of Efficient-B7 network across initial conditions. When the best CNNs are picked and ensembled the ensemble performance (Best_6_avg) reaches $1-score\le 0.924$. In the case of DeiT models, the ensemble was carried out without picking the best model across different DeiTs but still reaches similar classification accuracy (with the $F1-score$ reaching 0.924) with no hyperparameter tuning.”

Additionally, under the subheading ‘Why DeiT models ensemble better’,

“The CNN ensemble has more RRR cases (2523) than the EDeiT (2430), but when the three models have some disagreement, the EDeiTs catch up and outperform the CNN ensembles. In particular:

- The correct RWW cases are $2.3\times$ to $2.6\times$ more common in the geometric average and arithmetic average EDeiT respectively (Geometric CNN: 10, Geometric EDeiT: 23; Arithmetic CNN: 8, Arithmetic EDeiT: 21). In the SI (See Footnote 1) we show that the probability that a RWW ensembling results in a correct prediction depends on the ratio between the second and third component of the ensembled confidence vector, and that the better performance of DeiT ensembles in this situation is justified by the shape of the confidence vector.
- The correct RRW cases are $2.4\times$ more common in the geometric average and arithmetic average EDeiT (Geometric CNN: 93, Geometric EDeiT: 226; Arithmetic CNN: 94, Arithmetic DeiT: 225), and they represent the bulk of the improvement of DeiT versus CNN ensembles. Since the single-model performances are similar, this suggests that a higher degree of disagreement among individual models is allowing for a better ensembling.”

now reads:

“The CNN ensemble has more RRR cases (2523) than the EDeiT (2515), but when the three models have some disagreement, the EDeiTs catch up with the CNN ensembles. In particular:

- The correct RWW cases are $2.0\times$ more common in the geometric average and arithmetic average EDeiT (Geometric CNN: 8, Geometric EDeiT: 15; Arithmetic CNN: 8, Arithmetic EDeiT: 16). In the SI (See Footnote 1) we show that the probability that a RWW ensembling results in a correct prediction depends on the ratio between the second and third component of the ensembled confidence vector, and that the better performance of DeiT ensembles in this situation is justified by the shape of the confidence vector.

And,

“For DeiTs, we have $S=0.773\pm 0.004$, while for CNNs the similarity is much higher, $S=0.945\pm 0.003$.”

now reads:

“For DeiTs, we have $S=0.799\pm 0.004$, while for CNNs the similarity is much higher, $S=0.945\pm 0.003$.”

In addition,

“Given a fixed budget of single-model correct answers, this has a double benefit: (i) the best ensembled performance is obtained by maximizing the number of RRW combinations with respect to the RRR combinations; (ii) RWW combinations result more likely in a correct answer when the two wrong answers are different (see SI (See Footnote 1)). The situation is analogous for geometric averaging (Fig. 2c), where we further note that there can be WWW models resulting in a correct prediction, because all the (wrong) top answers of each model can be vetoed by another model.”

now reads:

“Given a fixed budget of single-model correct answers, RWW combinations result more likely in a correct answer when the two wrong answers are different (see SI (See Footnote 1)). The situation is analogous for geometric averaging (Fig. 2c).”

**Figure 2:**
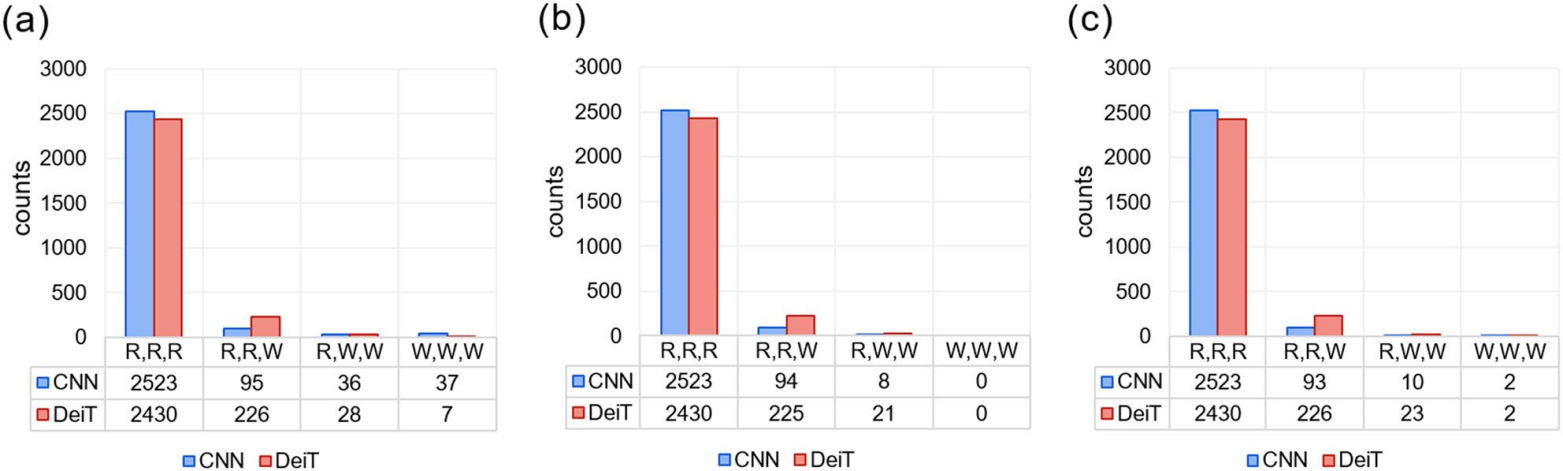
Comparison between three-model ensemble models based on CNNs and on DeiTs on the ZooLake test set. The bar heights indicate how often each combination (RRR, RRW, RWW, WWW) appeared. RRR indicates that all the models gave the right answer, RRW means that one model gave a wrong answer, and so on. The numbers below each bar indicate explicitly the height of the bar. On panel (**a**) we consider the whole test set, on panel (**b**) we only consider the examples which were correctly classified by the *arithmetic* ensemble average, and on panel (**c**) those correctly classified through *geometric* ensemble average.

In Table 1, the values for “Arithmetic ensemble (accuracy/F1-score)” and “Geometric ensemble (accuracy/F1-score)” were incorrect for the Model “DeiT-Base”. The correct and incorrect values appear below.

Incorrect:

**Table:** 

Model	Arithmetic ensemble (accuracy/F1-score)	Geometric ensemble (accuracy/F1-score)
DeiT-Base	0.994/0.973	0.996/0.984

Correct:

**Table:** 

Model	Arithmetic ensemble (accuracy/F1-score)	Geometric ensemble (accuracy/F1-score)
DeiT-Base	0.973/0.924	0.972/0.922

In the Discussion section,

“Besides being of simple training and deployment (we performed no specific tuning for any of the datasets), EDeiTs systematically lead to a substantial improvement in classifying biodiversity images across all tested datasets, when compared to the previous state of the art. Furthermore, our results were obtained by averaging over three DeiT models but increasing the number of individual learners can lead to a further improvement in the performances.”

now reads:

“Besides being of simple training and deployment (we performed no specific tuning for any of the datasets), EDeiTs achieve results comparable to those of earlier carefully tuned state-of-the-art methods, and even outperform them in classifying biodiversity images in four of the ten datasets.”

Finally, the Supplementary Information file published with this Article contained errors in the Supplementary Text, Tables 1, 2 and 3, as well as Figs. 4, 6 and 7. The original Supplementary Figs. 1 and 2 have been removed. The original Supplementary Information file is provided below.

The original Article and accompanying Supplementary Information file have been corrected.

## Supplementary Information

Supplementary Information.

