# Supplementary material for: Author Correction: Ensembles of data-efficient vision transformers as a new paradigm for automated classification in ecology
Source: Sci Rep. 2023 Apr 17;13:6243. doi: 10.1038/s41598-023-32258-4 (PMC10110596; doi:10.1038/s41598-023-32258-4)
Supplement: Supplementary file 1 — Supplementary Information. [file 41598_2023_32258_MOESM1_ESM.pdf]

Supplemental Information

S. Kyathanahally[1,*], T. Hardeman[1], M. Reyes[1], E. Merz[1], T. Bulas[1], P. Brun[2], F. Pomati[1], and M. Baity-Jesi[1,+]

[1]Eawag, Überlandstrasse 133, CH-8600 Dübendorf, Switzerland
[2]WSL, Zürcherstrasse 111, CH-8903 Birmensdorf, Switzerland
[*]sreenath.kyathanahally@eawag.ch
[+]marco.baityjesi@eawag.ch

ABSTRACT

Supplemental Information to *Ensembles of Data-efficient Vision Transformers as a New Paradigm for Automated Classification in Ecology*.

1 Comparisons of DeiT ensembles with previous SOTA

Here, we provide further comparisons between EDeiTs and the previous SOTA. In Tab. 1 and Tab. 2 we show both the model accuracy and the (macro) F1-score, in order to have both micro- and macro-averaged descriptors (*i.e.* descriptors which are directly influenced by data imbalance and that are not). Both improve systematically with respect to the SOTA. In particular, the improvement in the F1 scores indicates that the classification of the minority classes improved. Since the amount of improvement from the SOTA is bounded by the error of the SOTA, we find that a better metric to define the improvement of our models is the error, defined as the fraction of misclassified examples (*i.e.* 1-accuracy). As shown in the rightmost part of Tab. 1, for average ensembling the error is reduced widely, from a 18.48% decrease in the NA-Birds dataset (where the SOTA models are a kind of ViTs, TransFG[1]), to as much of an 87.5% error reduction in the RSMAS dataset, depicting corals (where the SOTA models are ensembles of convolutional networks). The performance increase in both accuracy and F1 score is even sharper when using geometric averaging (Tab. 2), where the error reduction ranges from 29.35% to 100%. A 100% error reduction means that none of the test images were misclassified by the EDeiT.

2 Performances on ZooLake

In Fig. 1 we show the per-class performances of the ensembled DeiT models on the ZooLake dataset. The model misclassified 15 out of 2691 test images. Of those, 14 belonged to junk/container categories that are not univocally defined (*i.e.* there is a degree of arbitrariness when defining and labeling the junk classes). In Fig. 2 we explicitly show the 15 misclassified images.

3 Arithmetic Average Ensembling in the RWW case

Here, we show that also when the correct guesses are not a majority, DeiT models tend to exhibit better arithmetic average ensembling. In Fig. 3 we show the sorted average confidence vector of the architectures we are considering. We see that all CNNs have a confidence profile which is significantly different from the DeiTs. We can thus assume that CNNs and DeiTs have two different confidence profiles, and call them $\vec{C}_{\mathrm{CNN}}$ and $\vec{C}_{\mathrm{DeiT}}$. The first component of these vectors, the largest one, C_0, indicates the class that is guessed by the model.

Since these vectors are highly peaked on C_0 (so $C_0 - C_1$ is large), we can state that, if an example is misclassified, then the single-model guesses will most likely be:

- All wrong (W,W,W). Since $C_0 - C_1$ is large, we cannot get a right ensemble prediction if all the models are wrong. This is true for all architectures, so this case cannot explain the better ensembling of the DeiTs.

- One right, and two wrong, with both wrong models guessing the same class (R,W_1,W_1). If both wrong guesses fall on the same class, the ensembled guess is wrong with high probability. Given the higher similarity among CNN confidences [Eq. (1) in the main text], we expect this situation to be more rare for DeiT models. We show this in Fig. 4a, where we see that the (R,W_1,W_1) cases are almost 8 times more common in the CNNs. Comparing with Fig. 4b, we see that most of these cases result in a mistake from the classifier. Therefore, the lower similarity between independent DeiT predictions implies better ensembling also in the (R,W_1,W_1) case.

Table 1. Summary of the performances of EDeiTs (combining learners through an arithmetic mean) and the current SOTA models on public datasets.

Dataset name	Image types	Classes	No. of images	Previous State of the Art			Arithmetic average EDeiTs			Absolute improvement		Relative improvement
				Accuracy	F1 Score	Error	Accuracy	F1-Score	Error	Accuracy	F1-Score	Error
RSMAS[2]	Coral-reef	14	766	0.992[3]	0.995	0.008	0.999	0.999	0.001	0.70%	0.40%	-87.50%
EILAT[2]	Coral-reef	8	1123	0.989[3]	0.990	0.011	0.997	0.997	0.003	0.80%	0.70%	-72.73%
ZooLake[4]	Plankton	35	17943	0.979[5]	0.927	0.021	0.994	0.973	0.006	1.50%	4.60%	-71.43%
WHOI [6]	Plankton	22	6600	0.961[5]	0.961	0.039	0.995	0.995	0.005	3.40%	3.40%	-87.18%
Kaggle [7]	Plankton	38	14374	0.947[5]	0.937	0.053	0.986	0.985	0.014	3.90%	4.80%	-73.58%
ZooScan [8]	Plankton	20	3771	0.898[5]	0.915	0.102	0.965	0.975	0.035	6.70%	6.00%	-65.69%
NA-Birds [9]	Birds	555	48562	0.908[1]	-	0.092	0.925	0.906	0.075	1.70%	-	-18.48%
Stanford Dogs [10]	Dogs	120	20580	0.923[1]	-	0.077	0.961	0.958	0.039	3.80%	-	-49.35%
Sri Lankan Beetles [11]	Tiger beetles	9	361	0.910 [11]	-	0.090	0.932	0.919	0.068	2.20%	-	-24.44%
Florida Wild trap [12]	Wild animals & birds	22	104495	0.790 [12]	-	0.210	0.928	0.613	0.072	13.80%	-	-65.71%

Table 2. Summary of the performances of EDeiTs (combining learners through geometric mean) and the current SOTA models on public datasets.

Dataset name	Image types	Classes	No. of images	Previous State of the Art			Geometric average EDeiTs			Absolute improvement		Relative improvement
				Accuracy	F1 Score	Error	Accuracy	F1-Score	Error	Accuracy	F1-Score	Error
RSMAS[2]	Coral-reef	14	766	0.992[3]	0.995	0.008	1.000	1.000	0.000	0.80%	0.50%	-100.00%
EILAT[2]	Coral-reef	8	1123	0.989[3]	0.990	0.011	0.997	0.997	0.003	0.80%	0.70%	-72.73%
ZooLake[4]	Plankton	35	17943	0.979[5]	0.927	0.021	0.996	0.984	0.003	1.80%	6.00%	-80.9%
WHOI [6]	Plankton	22	6600	0.961[5]	0.961	0.039	0.995	0.995	0.005	3.40%	3.40%	-87.18%
Kaggle [7]	Plankton	38	14374	0.947[5]	0.937	0.053	0.990	0.989	0.010	4.30%	5.20%	-81.13%
ZooScan [8]	Plankton	20	3771	0.898[5]	0.915	0.102	0.976	0.984	0.024	7.80%	6.90%	-76.47%
NA-Birds [9]	Birds	555	48562	0.908[1]	-	0.092	0.935	0.918%	0.065	2.70%	-	-29.35%
Stanford Dogs [10]	Dogs	120	20580	0.923[1]	-	0.077	0.972	0.970	0.028	4.90%	-	-63.64%
Sri Lankan Beetles [11]	Tiger beetles	9	361	0.910 [11]	-	0.090	0.932	0.912	0.068	2.20%	-	-24.44%
Florida Wild trap [12]	Wild animals & birds	22	104495	0.790 [12]	-	0.210	0.932	0.627	0.068	14.20%	-	-67.62%

- One right, and two wrong, with the two wrong guesses being on different classes (R, W_1, W_2). As shown in Fig. 4a, this occurs slightly more often with DeiTs, which is reasonable, given that DeiTs are more likely than CNNs to provide three different predictions. As we will show, in this situation, the probability that the classifier gives the correct answer depends on the shape of the confidence vector, and that of the DeiT gives better performances.

How the confidence vectors influence ensembling

We now show how the shape of the sorted confidence vectors is influences ensembling in the (R, W_1, W_2) case. As shown in Fig. 3, the confidence profiles $\vec{C}$ vary depending on the model class. Since the confidence vector does not have the same exact profile for every image the model sees, we assume that these are Gaussian and define a standard deviation (not standard error) vector $\vec{\sigma}$, which defines how much each component of $\vec{C}$ fluctuates around its central value.

The components of $\vec{C}$ indicate the probability that the model assigns to the classes. These roughly correlate with the true probabilities. We see this from Fig. 5a, which shows the top-k accuracy, A_k, as a function of k. A_k is the accuracy that we get if we define that a prediction is correct if any of the top k predictions is correct. We see that A_k starts at a value similar to C_0, and quickly grows, reaching 1 for small k.

In other words, when C_0 indicates the wrong class, it is most likely that C_1 is the correct class, and so on.

Let us call m_0, m_1 and m_2 the three models that are used for ensembling. The confidence profiles of these three models, which we will call $\vec{c}^0, \vec{c}^1$ and $\vec{c}^2$, will follow the Gaussian process defined by $\vec{C}$ and $\vec{\sigma}$, but the classes to which each of the confidences is assigned will vary according to the model. For concreteness, let us postulate that for a given image, depicting class A, m_0 provides a correct prediction. We can posit $\vec{c}^0 = \vec{C}$, and order the classes according to how they were scored by m_0. So, the confidence assigned to class A is $c^0(A) = C_0$ is the confidence assigned to class A by m_0; $c^0(B) = C_1$ is the confidence assigned to class B, and so on.

Let us now pass to m_1. Since by hypothesis it gives a wrong prediction, $c^1(A) \neq C_0$. Since when a model prediction is wrong, the second-ranked confidence is the most likely to be correct, typically $c^1(A) = C_1$. Model m_1 will assign C_0 to any of the remaining classes. In the most unfavorable cases, class B is very similar to class A, implying $c^1(B) = C_0$; and class C is also not too different, so $c^1(C) = C_2$. An equivalent reasoning for m_2 leads to $c^2(A) = C_1$, $c^2(B) = C_2$ and $c^2(C) = C_0$.

The predictions of the ensembled model are the averages across the three models,

$$c^{(\mathrm{ENS})}(A) = \frac{1}{3}(C_0 + 2C_1)\,, \tag{1}$$

$$c^{(\mathrm{ENS})}(B) = \frac{1}{3}(C_0 + C_1 + C_2)\,, \tag{2}$$

$$c^{(\mathrm{ENS})}(C) = \frac{1}{3}(C_0 + 2C_2)\,. \tag{3}$$

From Eqs. (1), (2) and (3) we see that in case of model disagreement, ensembling tends to choose the correct answer. However, it is also possible that because of the fluctuations of the confidences around their central value, $c^{(\mathrm{ENS})}(B)$ (and $c^{(\mathrm{ENS})}(C)$) become larger than $c^{(\mathrm{ENS})}(A)$, and the ensembled model misclassifies. The probability of a misclassification due to fluctuations is

$$P(c^{(\mathrm{ENS})}(A) < c^{(\mathrm{ENS})}(B)) = \frac{1}{2}\left[1 + \mathrm{erf}\left(\frac{C_2 - C_1}{\sqrt{2(2\sigma_0^2 + 3\sigma_1^2 + \sigma_2^2)}}\right)\right], \tag{4}$$

$$P(c^{(\mathrm{ENS})}(A) < c^{(\mathrm{ENS})}(C)) = \frac{1}{2}\left[1 + \mathrm{erf}\left(\frac{2(C_2 - C_1)}{\sqrt{2(2\sigma_0^2 + 2\sigma_1^2 + 2\sigma_2^2)}}\right)\right]. \tag{5}$$

Eqs. (4) and (5) reflect common intuition: the ensembled classifier is maximally efficient when the fluctuations are small and the difference $C_1 - C_2$ is big. Thus, to leading order, the comparison of the ensembled classifiers in the (R, W_1, W_2) situation boils down to a comparison of the ratio

$$R = \frac{(C_1 - C_2)}{\sqrt{2\sigma_0^2 + 3\sigma_1^2 + \sigma_2^2}} \tag{6}$$

related to each model. A larger R implies a lower error. For ensembles of CNNs, this ratio is $R^{(\mathrm{CNN})} = 0.08$, while for DeiT it is $R^{(\mathrm{DeiT})} = 0.10$, which translate into $P^{(\mathrm{CNN})} = 0.53$ and $P^{(\mathrm{DeiT})} = 0.54$. This is a small difference, but the main point is that it goes in the same direction of the (R, W_1, W_1) contribution.

4 Comparing DeiT with ViT

We compare DeiT with ViT models on the ZooLake dataset. In Tab. 3 we compare the performance of ViT with DeiT models. When we take the single models, DeiTs do not perform better than ViTs. In particular, the single-model performance of ViT-B16 models outperforms DeiTs. However, when ensembling is carried out, DeiTs perform much better, both in accuracy and in F1-score. In addition to ensembling ViTs over initial conditions, we also take the best ViT models from each of the architectures and make a 3-model ensemble (ViT_3_avg) over those (Tab. 3). The ensemble model with 3 different architectures is similar to performance obtained by ensembling three B-16 models. If we ensemble 5 models (three B16, one B32 and one L32) models then the F1-score slightly improves to 0.930 compared to 0.922 of ViT_3_avg. However, the ensemble of three DeiTs outperforms even the ensemble of 5 ViTs.

This better generalization stems from a major mutual independence of individual learners. This can be seen from the similarity between confidence vectors [Eq. (1) in the main text] of ViTs versus DeiTs. While for DeiTs we have $S = 0.773 \pm 0.004$, the similarity of ViTs is much higher ($S = 0.969 \pm 0.002$ for ViT-B16, $S = 0.955 \pm 0.003$ for ViT-B32, $S = 0.954 \pm 0.003$ for ViT-L32, and $S = 0.956 \pm 0.003$ for the ensemble over different ViT architectures).

This results in the ViT ensembles being more often in a (RRR) configuration, as shown in Fig. 6–left. However, the higher number of (RRR) is overcompensated by a lower number of (RRW) [and of correctly classified (RWW), Fig. 6], which eventually result in the DeiT ensemble having a better performance.

This is also seen in the higher rate of correctly classified (RWW) examples of DeiTs with respect to ViTs (Fig. 7), analogously to what we showed in App. 3 for CNNs. Analogous considerations also apply here, with the confidence vectors being qualitatively dissimilar from DeiTs (and similar to CNNs) also in the case of ViTs (Fig. 8).

Table 3. Summary of the performance of the individual models on the ZooLake dataset. The ensemble score on the rightmost column is obtained by averaging across 3 different initial conditions. The ViT_3_avg model is an ensemble of the best of each ViT-B16, ViT-B32 & ViT-L32 models . The numbers in parentheses are the standard errors, referred to the last significant digit.

Type	No. of params for each model	Accuracy Mean	F1-score Mean	Arithmetic Average Ensemble (accuracy/ F1-score)	Geometric Average Ensemble (accuracy/ F1-score)
**ViT-B16**	85.7M	0.973(1)	0.918(2)	0.976/0.919	0.976/0.921
**ViT-B32**	87.5M	0.960(2)	0.886(6)	0.966/0.893	0.964/0.889
**ViT-L32**	305.5M	0.960(2)	0.894(2)	0.967/0.908	0.966/0.903
**ViT_3_avg**	-	-	-	0.972/0.922	0.974/0.931
**DeiT-Base**	85.8M	0.962(3)	0.899(2)	0.994/0.973	0.996/0.984

References

1. He, J. *et al.* Transfg: A transformer architecture for fine-grained recognition. *CoRR* **abs/2103.07976** (2021). 2103.07976.
2. Gómez-Ríos, A. *et al.* Towards highly accurate coral texture images classification using deep convolutional neural networks and data augmentation. *Expert. Syst. with Appl.* **118**, 315–328, DOI: https://doi.org/10.1016/j.eswa.2018.10.010 (2019).
3. Lumini, A., Nanni, L. & Maguolo, G. Deep learning for plankton and coral classification. *Appl. Comput. Informatics* (2020).
4. Kyathanahally, S. *et al.* Data for: Deep learning classification of lake zooplankton, DOI: 10.25678/0004DY (2021).
5. Kyathanahally, S. P. *et al.* Deep learning classification of lake zooplankton. *Front. microbiology* 3226, DOI: 10.3389/fmicb.2021.746297 (2021). arXiv:2108.05258.
6. Sosik, H. & Olson, R. Automated taxonomic classification of phytoplankton sampled with imaging-in-flow cytometry. *Limnol. Oceanogr.* **5**, 204–216 (2007).
7. Zheng, H. *et al.* Automatic plankton image classification combining multiple view features via multiple kernel learning. *BMC Bioinforma.* **18**, 570, DOI: 10.1186/s12859-017-1954-8 (2017).
8. Gorsky, G. *et al.* Digital zooplankton image analysis using the ZooScan integrated system. *J. Plankton Res.* **32**, 285–303, DOI: 10.1093/plankt/fbp124 (2010). https://academic.oup.com/plankt/article-pdf/32/3/285/4394627/fbp124.pdf.
9. Van Horn, G. *et al.* Building a bird recognition app and large scale dataset with citizen scientists: The fine print in fine-grained dataset collection. In *2015 IEEE Conference on Computer Vision and Pattern Recognition (CVPR)*, 595–604, DOI: 10.1109/CVPR.2015.7298658 (2015).
10. Khosla, A., Jayadevaprakash, N., Yao, B. & Fei-Fei, L. Novel dataset for fine-grained image categorization. In *First Workshop on Fine-Grained Visual Categorization, IEEE Conference on Computer Vision and Pattern Recognition* (Colorado Springs, CO, 2011).
11. Abeywardhana, D., Dangalle, C., Nugaliyadde, A. & Mallawarachchi, Y. Deep learning approach to classify tiger beetles of Sri Lanka. *Ecol. Informatics* **62**, 101286, DOI: https://doi.org/10.1016/j.ecoinf.2021.101286 (2021).
12. Gagne, C., Kini, J., Smith, D. & Shah, M. Florida wildlife camera trap dataset. *CoRR* **abs/2106.12628** (2021). 2106.12628.

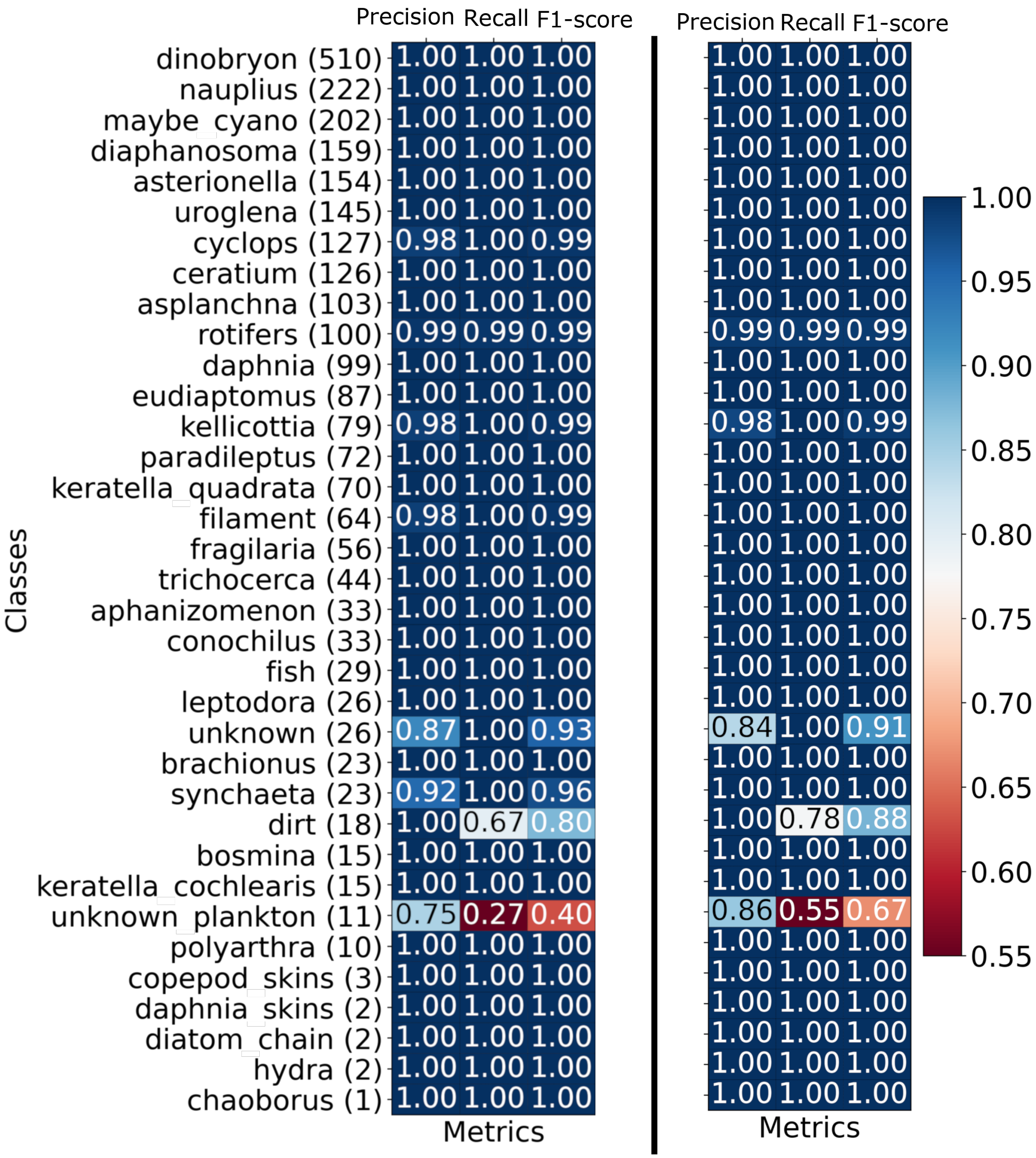

Figure 1. Per-Class precision, recall and F1-score of EDeiT models on the ZooLake dataset. On the left side, we have the arithmetic average EDeiT, and on the right we have the geometric average. The number within brackets indicates the number of test images of the corresponding class.

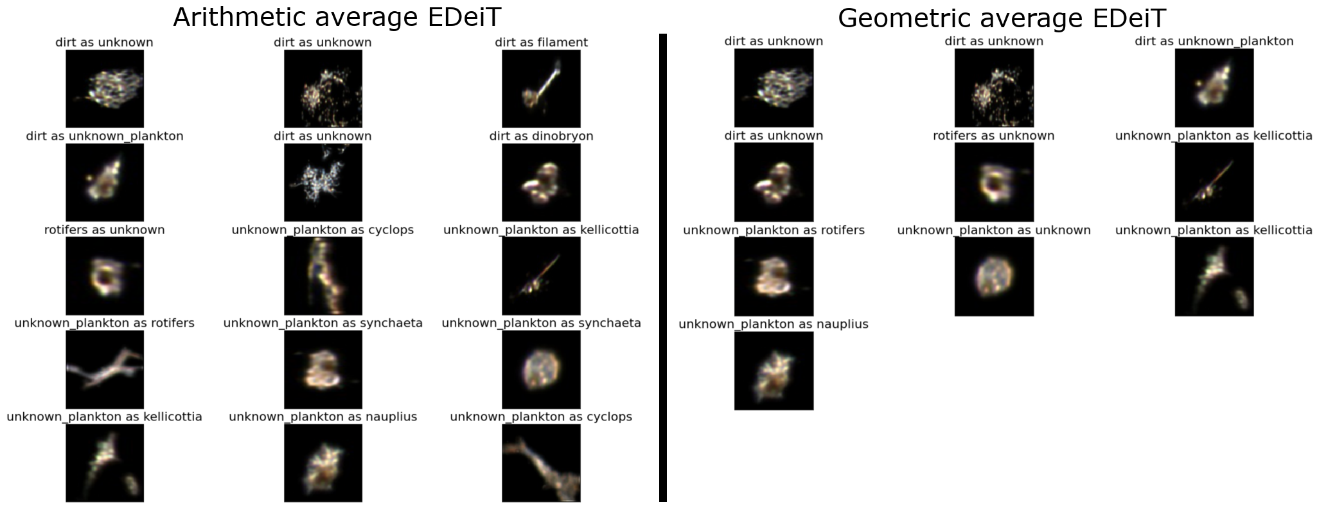

Figure 2. Misclassified images of the EDeiT models on the ZooLake dataset. On the left side, we have the misclassifications from arithmetic average EDeiT, and on the right from geometric average EDeiT. All the misclassifications involve junk classes and all the images misclassified by Geometric average EDeiT were also misclassified by arithmetic average EDeiT.

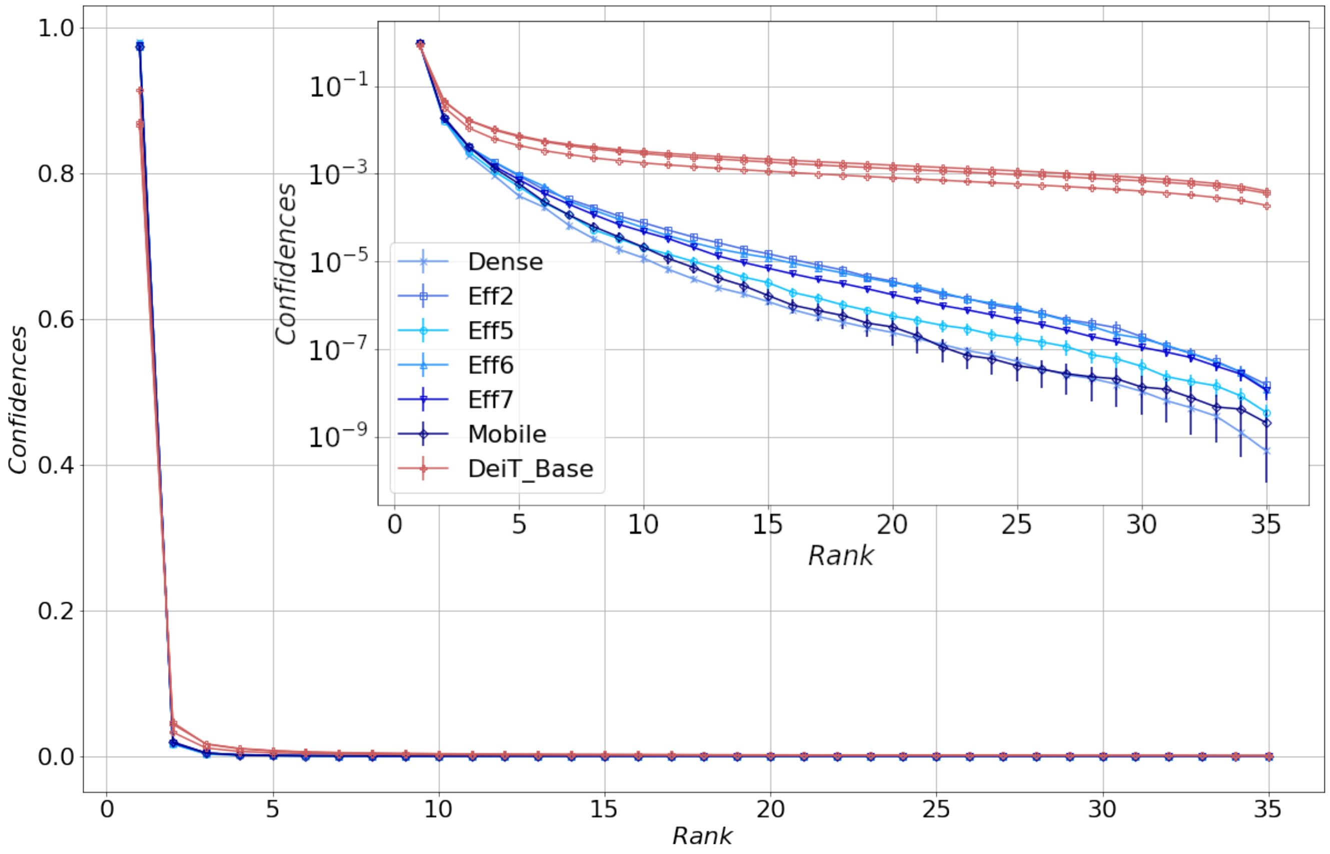

Figure 3. For various model types, the mean confidences (y-axis) across all classes (x-axis) are shown. When it comes to classification, the CNN models (blues) are more confident than the DeiT models (red), as can be observed in the outset figure (linear scale). The inset figure shows the same in log scale.

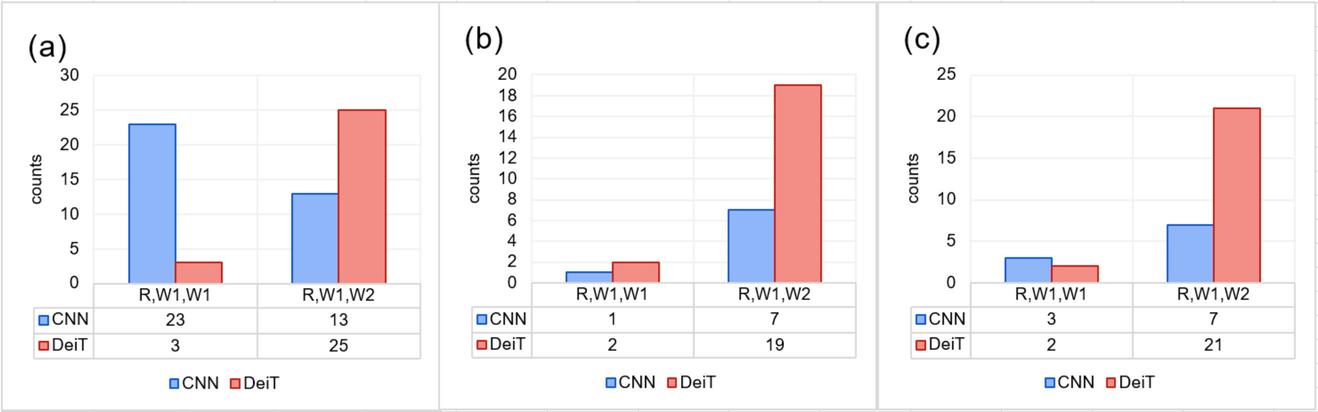

Figure 4. Comparison between ensembled CNNs (EfficientNet B7, MobileNet, DenseNet) with ensembled DeiTs. (**a**): The bars show how many times, out of the full ZooLake test set, the single learners within the ensemble model gave one correct answer (R) and two wrong answers that were indicating the same class (W_1 and W_1). The bars indicate one R answer and two wrong answers that differ from each other (W_1 and W_2). (**b**): Same as (a), but only restricted to the examples that resulted in a correct classification by the arithmetic average EDeiT model. (**c**): Same as (a), but only restricted to the examples that resulted in a correct classification by the geometric average EDeiT model.

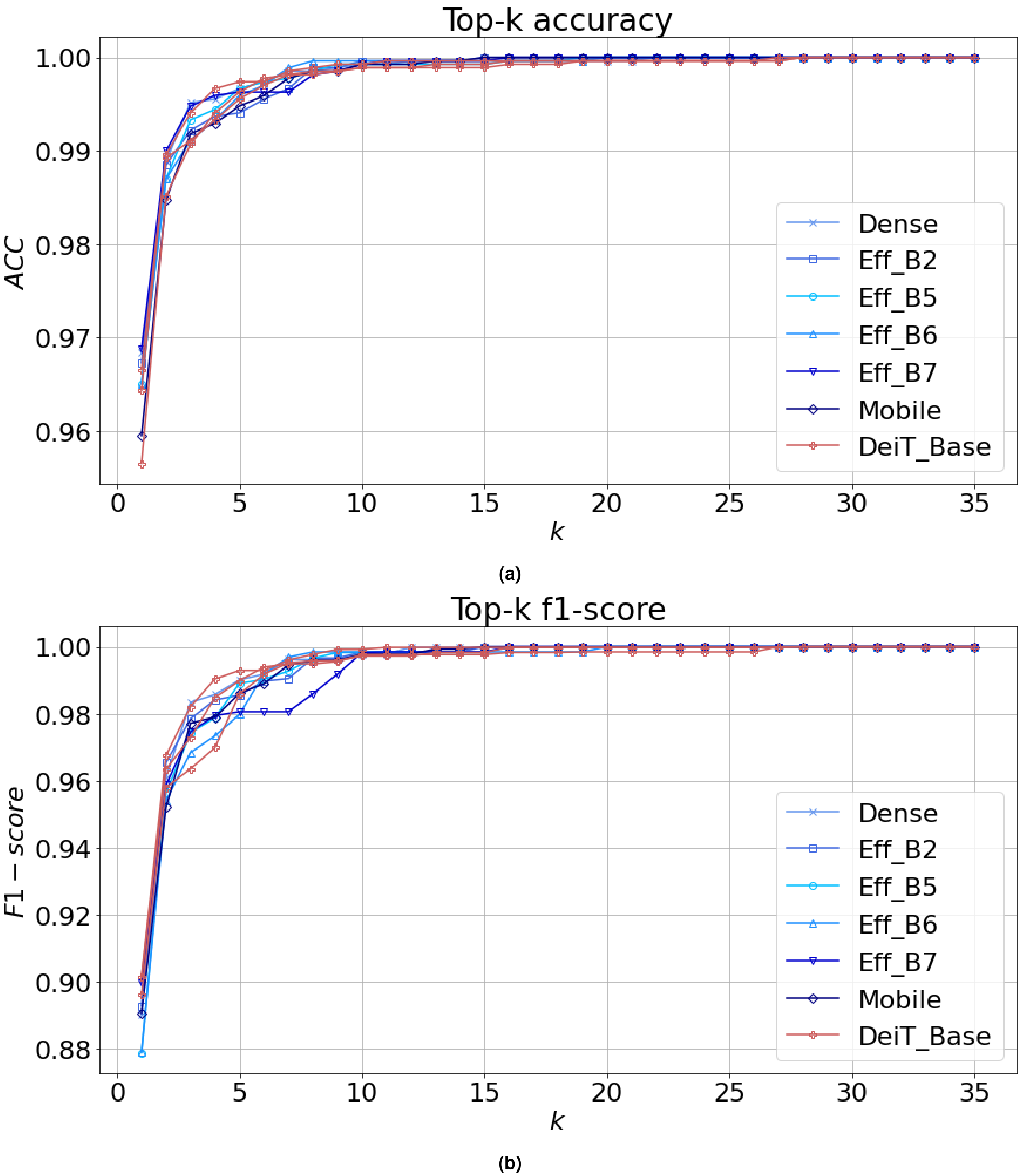

Figure 5. (a) Top-*k* accuracy vs *k* and (b) Top-*k* f1-score vs *k* for the several models tested on the ZooLake dataset.

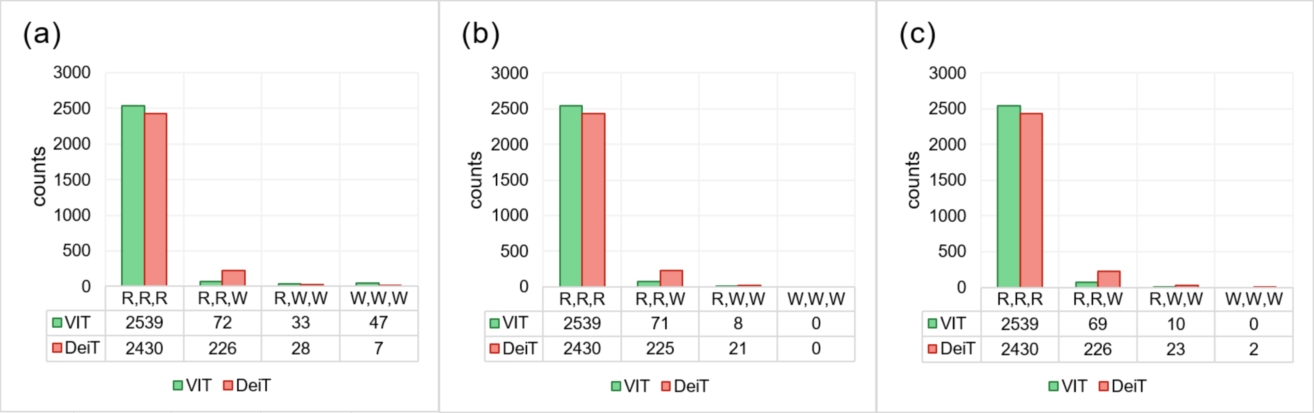

Figure 6. Comparison between 3-model ensemble models based on VITs (B16, B32 and L32) and on DeiTs on the ZooLake test set. The bar heights indicate how often each combination (RRR, RRW, RWW, WWW) appeared. RRR indicates that all the models gave the right answer, RRW means that one model gave a wrong anwswer, and so on. The numbers below each bar indicate explicitly the height of the bar. On panel **(a)** we consider the whole test set, on panel **(b)** we only consider the examples which were correctly classified by the arithmetic average EDeiT model and on panel **(c)** we only consider the examples which were correctly classified by the geometric average EDeiT model.

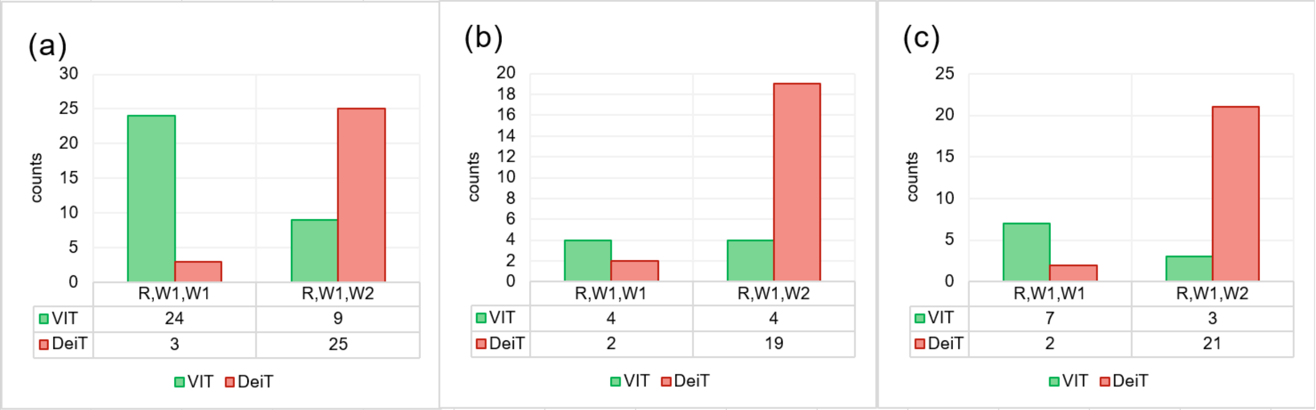

Figure 7. Comparison between ensembled VITs (B16, B32, L32) with ensembled DeiTs. (**a**): The bars show how many times, out of the full ZooLake test set, the single learners within the ensemble model gave one correct answer (R) and two wrong answers that were indicating the same class (W_1 and W_1). The bars indicate one R answer and two wrong answers that differ from each other (W_1 and W_2). (**b**): Same as (a), but only restricted to the examples that resulted in a correct classification by the arithmetic average EDeiT model. (**c**): Same as (a), but only restricted to the examples that resulted in a correct classification by the geometric average EDeiT model.

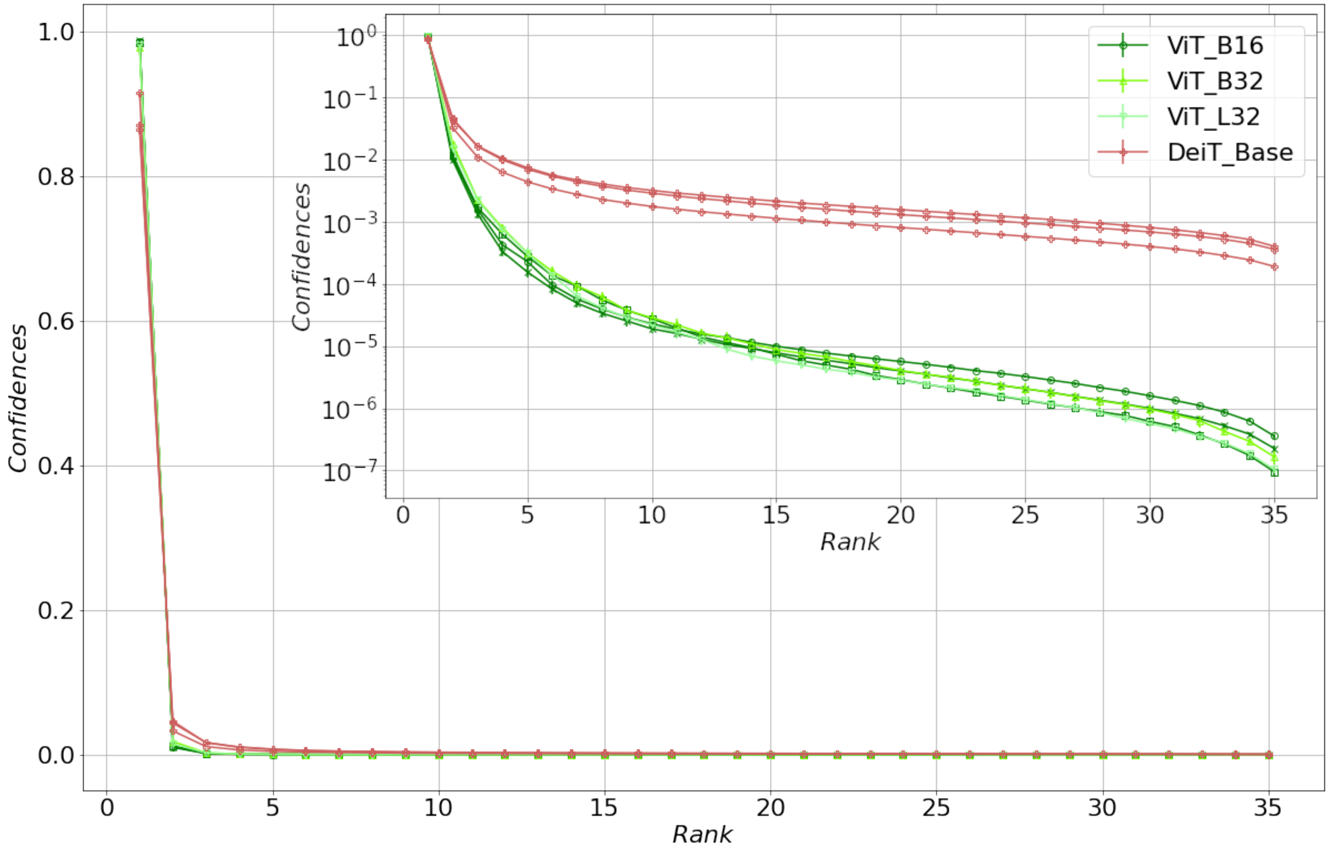

Figure 8. For various model types, the mean confidences across all classes (x-axis) are shown. When it comes to classification, the VIT models (greens) are more confident than the DeiT-base models (red), as can be observed in the outset figure (linear scale). The inset figure shows the same in log scale.
